# Supplementary material for: Genetic and clinical determinants of neonatal jaundice and growth patterns in the Qingdao birth cohort: A genome-wide association study
Source: PLoS One. 2026 Jan 16;21(1):e0338567. doi: 10.1371/journal.pone.0338567 (PMC12810793; doi:10.1371/journal.pone.0338567)
Supplement: S1 File — (DOCX) [file pone.0338567.s018.docx]

URLs

Variant Effect Predictor (VEP): https://asia.ensembl.org/info/docs/tools/vep/script/vep_download.html

GWAS catalog database: https://www.ebi.ac.uk/gwas/downloads

Liftover: http://hgdownload.cse.ucsc.edu/admin/exe/ linux.x86_64/liftOver

Locuszoom: http://locuszoom.sph.umich.edu

Plink (v1.9): http://www.cog-genomics. org/plink/1.9/

EIGENSOFT (v7.2.1): https://anaconda.org/bioconda/eigensoft

DAVID: https://david.ncifcrf.gov

GTEx: https://www.gtexportal.org/home/

BioBank Japan (BBJ): https://humandbs.biosciencedbc.jp/files/hum0197/
