# Supplementary material for: Genetic and clinical determinants of neonatal jaundice and growth patterns in the Qingdao birth cohort: A genome-wide association study
Source: PLoS One. 2026 Jan 16;21(1):e0338567. doi: 10.1371/journal.pone.0338567 (PMC12810793; doi:10.1371/journal.pone.0338567)
Supplement: Attachment — Submitted filename: review-PONE-D-25-10541.docx [file pone.0338567.s019.docx]

Comments to the Author

Thank you for submitting this paper for review. Please, find my comments below:

1. The wording /English language needs improving throughout.
2. The opening statement, "The health and development of newborns are always a concern," is vague. Please specify the current research status and significance of neonatal jaundice and growth patterns.
3. The Introduction lacks epidemiological or clinical context (e.g., global/Chinese incidence rates, risk factors for neonatal jaundice). Please supplement with relevant data.
4. Exclusion criteria for confounders (e.g., preterm birth, maternal diseases) are not described in the Methods. Please clarify inclusion/exclusion criteria.
5. Seven novel missense mutations (e.g., rs1042597) lack functional predictions (e.g., SIFT/PolyPhen-2 scores) or conservation analyses in the Results. Please provide bioinformatics support.
6. Functional validation (e.g. in vitro studies) or larger cohort replication is recommended if available.
7. Study limitations (e.g., regional bias from Qingdao samples) are not addressed. Please acknowledge generalizability constraints.
8. The Conclusion repeats results but does not highlight core contributions (e.g., "First identification of X novel SNPs in Chinese neonates"). Please succinctly summarize innovations.
9. Please outline future research.
