# Supplementary material for: Genetic and clinical determinants of neonatal jaundice and growth patterns in the Qingdao birth cohort: A genome-wide association study
Source: PLoS One. 2026 Jan 16;21(1):e0338567. doi: 10.1371/journal.pone.0338567 (PMC12810793; doi:10.1371/journal.pone.0338567)
Supplement: Attachment — Submitted filename: Response to Reviewers.docx [file pone.0338567.s020.docx]

**Reply to Reviewer Comments for Manuscript PONE-D-25-10541，**

**“Genetic and Clinical Determinants of Neonatal Jaundice and Growth Patterns in the Qingdao Birth Cohort: A Genome-Wide Association Study”**

**Editor：**

1. Please ensure that your manuscript meets PLOS ONE's style requirements, including those for file naming. The PLOS ONE style templates can be found at https://journals.plos.org/plosone/s/file?id=wjVg/PLOSOne_formatting_sample_main_body.pdf and https://journals.plos.org/plosone/s/file?id=ba62/PLOSOne_formatting_sample_title_authors_affiliations.pdf

**Responses:**

Thank you for your feedback. We have revised the formatting of our manuscript based on the reference material you provided.

2. Your ethics statement should only appear in the Methods section of your manuscript. If your ethics statement is written in any section besides the Methods, please delete it from any other section.

**Responses:**

We have removed the ethical statement from the Declarations section.

3. We note that Figure 4 in your submission contain [map/satellite] images which may be copyrighted. All PLOS content is published under the Creative Commons Attribution License (CC BY 4.0), which means that the manuscript, images, and Supporting Information files will be freely available online, and any third party is permitted to access, download, copy, distribute, and use these materials in any way, even commercially, with proper attribution. For these reasons, we cannot publish previously copyrighted maps or satellite images created using proprietary data, such as Google software (Google Maps, Street View, and Earth). For more information, see our copyright guidelines: http://journals.plos.org/plosone/s/licenses-and-copyright.

We require you to either (1) present written permission from the copyright holder to publish these figures specifically under the CC BY 4.0 license, or (2) remove the figures from your submission:

a. You may seek permission from the original copyright holder of Figure 4 to publish the content specifically under the CC BY 4.0 license.

We recommend that you contact the original copyright holder with the Content Permission Form (http://journals.plos.org/plosone/s/file?id=7c09/content-permission-form.pdf) and the following text:

“I request permission for the open-access journal PLOS ONE to publish XXX under the Creative Commons Attribution License (CCAL) CC BY 4.0 (http://creativecommons.org/licenses/by/4.0/). Please be aware that this license allows unrestricted use and distribution, even commercially, by third parties. Please reply and provide explicit written permission to publish XXX under a CC BY license and complete the attached form.”

Please upload the completed Content Permission Form or other proof of granted permissions as an "Other" file with your submission.

In the figure caption of the copyrighted figure, please include the following text: “Reprinted from [ref] under a CC BY license, with permission from [name of publisher], original copyright [original copyright year].”

b. If you are unable to obtain permission from the original copyright holder to publish these figures under the CC BY 4.0 license or if the copyright holder’s requirements are incompatible with the CC BY 4.0 license, please either i) remove the figure or ii) supply a replacement figure that complies with the CC BY 4.0 license. Please check copyright information on all replacement figures and update the figure caption with source information. If applicable, please specify in the figure caption text when a figure is similar but not identical to the original image and is therefore for illustrative purposes only.

The following resources for replacing copyrighted map figures may be helpful:

USGS National Map Viewer (public domain): http://viewer.nationalmap.gov/viewer/

The Gateway to Astronaut Photography of Earth (public domain): http://eol.jsc.nasa.gov/sseop/clickmap/

Maps at the CIA (public domain): https://www.cia.gov/library/publications/the-world-factbook/index.html and https://www.cia.gov/library/publications/cia-maps-publications/index.html

NASA Earth Observatory (public domain): http://earthobservatory.nasa.gov/

Landsat: http://landsat.visibleearth.nasa.gov/

USGS EROS (Earth Resources Observatory and Science (EROS) Center) (public domain): http://eros.usgs.gov/#

Natural Earth (public domain): <http://www.naturalearthdata.com/>

**Responses:**

We have removed the map and replaced it with a new figure to present the same analysis results.

4. We notice that your supplementary figures are uploaded with the file type 'Figure'. Please amend the file type to 'Supporting Information'. Please ensure that each Supporting Information file has a legend listed in the manuscript after the references list.

**Responses:**

We have added legends for the supplementary figures and supplementary tables after the references.

5. Please upload a copy of Supporting Information Table S1, S2, S3, S4, S5, S6, S7, S8, S9, S10, S11, which you refer to in your text on page 6, 7, 8, 9.

**Responses:**

We have uploaded the supplementary table.

**Reviewer #1:**

*Thank you for submitting this paper for review. Please, find my comments below:*

1. *The wording /English language needs improving throughout.*

**Responses:**

Thank you for your feedback. We have thoroughly revised the manuscript to improve its linguistic fluency throughout.

2. *The opening statement, "The health and development of newborns are always a concern," is vague. Please specify the current research status and significance of neonatal jaundice and growth patterns.*

**Responses:**

Thank you for your feedback. We revised the Background sentence in the abstract to more clearly reflect the clinical relevance and current research context.

3. *The Introduction lacks epidemiological or clinical context (e.g., global/Chinese incidence rates, risk factors for neonatal jaundice). Please supplement with relevant data.*

**Responses:**

Thank you for your feedback. We added a brief epidemiological and clinical context to the Introduction to clarify the frequency, clinical relevance, and major risk factors of neonatal jaundice.

4. *Exclusion criteria for confounders (e.g., preterm birth, maternal diseases) are not described in the Methods. Please clarify inclusion/exclusion criteria.*

**Responses:**

Thank you for your feedback. We have now clarified the exclusion of preterm cases.

Other maternal complications could not be systematically excluded due to incomplete records. These potential confounding factors need to be considered and controlled for in subsequent studies.

5*.* *Seven novel missense mutations (e.g., rs1042597) lack functional predictions (e.g., SIFT/PolyPhen-2 scores) or conservation analyses in the Results. Please provide bioinformatics support.*

**Responses:**

Thank you for your feedback. We have added functional annotation results for the seven novel missense variants based on SIFT and PolyPhen-2 predictions in the Results section and included the full data in a supplementary table (Table S14).

6. *Functional validation (e.g. in vitro studies) or larger cohort replication is recommended if available.*

**Responses:**

Thank you for the suggestion. We agree that functional validation and replication in additional cohorts would strengthen the findings. We have now added a description reflecting this point in the Discussion section.

7. *Study limitations (e.g., regional bias from Qingdao samples) are not addressed. Please acknowledge generalizability constraints.*

**Responses:**

Thank you for your feedback. We have now acknowledged the limitation of the regional source of the cohort and noted that caution is required when generalizing the findings to other populations.

8. *The Conclusion repeats results but does not highlight core contributions (e.g., "First identification of X novel SNPs in Chinese neonates"). Please succinctly summarize innovations.*

**Responses:**

We have revised the Conclusion to reduce repetition of detailed results and to more clearly highlight the main contributions of this study.

9. *Please outline future research.*

**Responses:**

We have outlined the directions for future research in the discussion, including the need for functional validation, replication in additional cohorts, and longitudinal follow-up to assess long-term outcomes.

**Reviewer #2:**

*I appreciate the opportunity to evaluate this timely study on neonatal growth genetics. The authors present a valuable GWAS dataset (n=6,685) with robust quality control, identifying both novel SNPs (e.g., rs148399850) and clinically relevant pathways (e.g., flavonoid glucuronidation). While the rigorous methodology and comprehensive functional enrichment analyses are clear strengths, I recommend clarifying several methodological points and contextualizing findings more deeply to enhance impact.*

*1.Methods*

*Please:*

*• Clarify if recruitment was random; address potential selection bias. Mention inclusion and exclusion criteria*

**Responses:**

Thank you for the comment. We have clarified in the Methods that newborns were randomly recruited; preterm infants and those who had recent blood transfusions were excluded.

*•* *State if multiple testing correction was applied beyond the GWAS threshold (P =1×10-8).*

**Responses:**

Thank you for the comment. Only the standard GWAS significance threshold (P < 5×10⁻⁸) was applied in this study, which is widely accepted to control the family-wise error rate (FWER). Because of the limited sample size, loci with P < 1×10⁻⁵ were also explored for functional relevance. Additional corrections such as FDR or Bonferroni can be added if required.

*• For SNPs with P =1×10-5add* *sensitivity analyses to reduce false positives.*

**Responses:**

Thank you for your helpful suggestion. We have conducted the leave-one-out sensitivity analysis (Fig S5), and added the description and statement in the revised manuscript.

*2. Results*

*Please:*

*• Resolve the contradiction between "no significant SNPs" and later reporting 109 SNPs (P =1×10-5).*

**Responses:**

Thank you for pointing this out. We have revised the results to distinguish significant loci from suggestive associations, clarifying the statistical thresholds and removing ambiguity.

*•* *The reported differences in allele frequencies between populations (e.g., Xizang vs. Guangxi) are interesting. A statistical test (e.g., χ²) could further strengthen these observations, though the trends remain biologically plausible.*

**Responses:**

Thank you for your helpful suggestion. We have now performed χ² tests to assess allele-frequency differences among populations. The corresponding results have been added as Table S8, and a brief description was included in the results section .

*3. Discussion*

*Please:*

*• Propose functional validation (e.g., in vitro assays for novel SNPs like rs927472313 in TMEM236).*

**Responses:**

Thank you for your helpful suggestion. We will conduct functional verification in our future research, and the corresponding paragraphs have been added to the Discussion section of the manuscript .

*• Limitations:*

*o Confounding factors (maternal nutrition, SES) were not adjusted for.*

*o Generalizability to non-East Asian populations is unclear.*

**Responses:**

Thank you for your insightful comments. Due to the limitations of our sampling design, we could not comprehensively control for all potential confounding factors. The confounders that were excluded have now been specified in the revised manuscript . We also acknowledge the regional limitation of our cohort, which should be addressed in future studies. Corresponding statements regarding confounding factors and regional generalizability have been added to the discussion section.
